# Experimental Studies of Active and Passive Flow Control Techniques Applied in a Twin Air-Intake

**DOI:** 10.1155/2013/523759

**Published:** 2013-06-26

**Authors:** Akshoy Ranjan Paul, Shrey Joshi, Aman Jindal, Shivam P. Maurya, Anuj Jain

**Affiliations:** ^1^Department of Applied Mechanics, Motilal Nehru National Institute of Technology Allahabad, Allahabad 211004, India; ^2^Department of Mechanical Engineering, Motilal Nehru National Institute of Technology Allahabad, Allahabad 211004, India; ^3^Department of Chemical Engineering, Motilal Nehru National Institute of Technology Allahabad, Allahabad 211004, India

## Abstract

The flow control in twin air-intakes is necessary to improve the performance characteristics, since the flow traveling through curved and diffused paths becomes complex, especially after merging. The paper presents a comparison between two well-known techniques of flow control: active and passive. It presents an effective design of a vortex generator jet (VGJ) and a vane-type passive vortex generator (VG) and uses them in twin air-intake duct in different combinations to establish their effectiveness in improving the performance characteristics. The VGJ is designed to insert flow from side wall at pitch angle of 90 degrees and 45 degrees. Corotating (parallel) and counterrotating (V-shape) are the configuration of vane type VG. It is observed that VGJ has the potential to change the flow pattern drastically as compared to vane-type VG. While the VGJ is directed perpendicular to the side walls of the air-intake at a pitch angle of 90 degree, static pressure recovery is increased by 7.8% and total pressure loss is reduced by 40.7%, which is the best among all other cases tested for VGJ. For bigger-sized VG attached to the side walls of the air-intake, static pressure recovery is increased by 5.3%, but total pressure loss is reduced by only 4.5% as compared to all other cases of VG.

## 1. Introduction

Twin air-intake ducts are widely used in aircrafts for the purpose of providing pressurized air to the air compressor of an aeroengine (i.e., gas turbine engine used in aircraft) in order to achieve sufficient thrust to accomplish necessary maneuvers. The importance of the air-intake can be understood from the fact that around 20% of the swept volume of the aircraft is required to be ingested by the air-intakes for the engine during normal cruise, while for climb and take-off, the proportions are even higher [1].

In order to increase the performance and to maintain the stability of an engine operation, air flow at the engine inlet face should be sufficiently decelerated, having low total pressure distortion, high uniformity of the flow with minimum cross-flow velocity components and swirl. Therefore, the task of air-intake is to maximize the static pressure recovery and the flow uniformity at the engine inlet face/compressor inlet called “aerodynamic inlet plane” (AIP). The static pressure is increased by making the air-intake long and diverging.

Twin-side air-intakes with Y-configuration are commonly used for ingesting atmospheric air to the engine of single-engine combat aircrafts. In such air-intakes, air is ingested from either side of the aircraft with its two individual S-shaped diverging limbs merging into a single diverging duct leading air to AIP. Curvature to the duct is provided to accommodate it in a smaller space. It is reported that inhomogeneous flow generated by the supersonic part of the air-intake causes flow separation and its subsonic part causes secondary flow due to centerline curvature of the air-intake. The S-bends forming the twin air-intake initiate the strong swirl which eventually manifests itself on formation of vortices and cross-stream pressure gradients.

The nonuniformity at the AIP causes an uneven impact loading at the downstream components, like compressor. Persistence of such condition may cause sudden failure of compressor parts (e.g., blades) during flight, which may lead to catastrophe. Therefore, these conditions are unacceptable from aerodynamic as well as structural viewpoints. Thus, good aerodynamic design of twin air-intake is a challenge to increase overall performance and stability of the aircraft by ensuring sufficient uniform air supply. Employing a passive flow control (in which no external energy or no additional mass is injected) or an active flow control techniques (in which external energy as well as additional mass is injected into the system) is the possible solutions to accomplish nearly uniform air supply. However, optimizing it for a wide range of speeds, altitudes, and maneuvers poses further challenges. 

 The surface-mounted vane-type submerged vortex generator (VG), which is an example of passive flow control device, is used on the internal surfaces of the twin air-intakes to mix the low-momentum boundary layer with a higher momentum core flow to help reduce or eliminate boundary layer separation. The microvortices generated by these VG arrays can also be used favourably to redirect secondary flows. In both cases, the goal is to improve the performance of the engine by increasing engine face pressure recovery and decreasing engine face pressure distortion.

 Several researchers [2–6] have contributed towards the effective design of VG. This VG is thin plate of triangular or trapezoidal shapes and is placed normal to the surface and at a lateral angle to the flow (referred to as inclination angle or vortex generator angle, **β**). Reichert and Wendt [7] used a low-profile “wishbone” type vortex generator to improve the total pressure distortions and recovery performance of a diffusing duct. The configuration employing the largest vortex generator was most effective in reducing distortion but did not produce major total pressure recovery. In a recent study, Paul et al. [8] showed the usefulness of fin-type submerged VG in flow improvement of an S-shaped diffusing duct. In another study, Paul et al. [9, 10] used similar VGs in a twin air-intake duct for flow control, especially at the AIP. The computational study demonstrated the efficacy of co-rotating VG array in reducing the flow distortion at the engine face. Johnston and Nishi [11] used a spanwise array of small, skewed, and pitched type of active flow control device called “vortex generator jets” (VGJs) in a turbulent boundary layer and proved the existence of longitudinal vortices downstream of the jet holes similar to the vortices behind the solid vortices. Johnston et al. [12] in another study described the development of vortex from VGJ. They performed experiment on a low-speed free-surface water channel to investigate the effect of dominance of VGJ array for its various configurations.

Lin [6] experimentally studied different types of vortex generating devices for turbulent flow separation control at low speeds. They used submerged vortex generators (wheeler doublet and wishbone type), spanwise cylinders, large eddy breakup (LEBU) device at small angle of attack, and vortex generator jets (VGJs).

 Sullerey and Pradeep [13] reported the effectiveness of VGJ in controlling secondary flows in rectangular S-shaped diffusing ducts (resemble to single-limb air-intake) having an area ratio (*A*_*r*_) of 1.39 and a turning angle (Δ*β*) of 21°/21°. The test was carried out for two inflow conditions: uniform and distorted. The use of VGJ resulted in over a 30% decrease in total pressure loss coefficient (*C*_TL_) and flow distortion coefficient (DC_60_). But for distorted inflow, a combination of passive device (tapered fin VG) and active device (VGJ) was used to reduce *C*_TL_ by 25%. 

 Harrison et al. [14] conducted experiments on boundary-layer-ingesting serpentine air-intake located on the aft surface of a blended-wing-body aircraft. Both suction and blowing (circumferential and reverse pyramid types) were applied at various locations in the air-intake in order to simulate the use of fluidic VGJ. The objective of using the VGJ was to redistribute the ingested low-momentum fluid around the periphery of the diffuser in order to normalize the flow distortion at the engine face, and thereby decreasing the fatigue and increasing operational surge margin. 

From the literature review, it is revealed that the active flow control technique is more robust and can be used in various flow fields as it gives an extra degree of freedom as compared to passive methods. This can be easily understood by the fact that in active flow control methodology, the parameter which controls the flow can be varied according to the flow field and is desirable. The passive flow control technique, like vane-type and solid VG, however, has other advantages, such as simplicity, ruggedness, and low cost. It has practical applications in stall control on airfoils and in diffusing ducts. But it has limitations such as it does not have the ability to provide a time-varying control action, whereas inserted VGJ could be time varying (rotating jets) and could be switched on/off or even an increase and decrease in magnitude of jet energy could be possible if desired. However, the comparative study of both techniques while applied in the flow control of twin air-intakes is missing in the current literature. 

 The objective of the present study is therefore to compare the two techniques of flow control (namely, passive and active) in a twin air-intake and to find out the efficacy of each of the techniques in terms of aerodynamic performance.

## 2. Experimental Methodology

**Figure 1:**
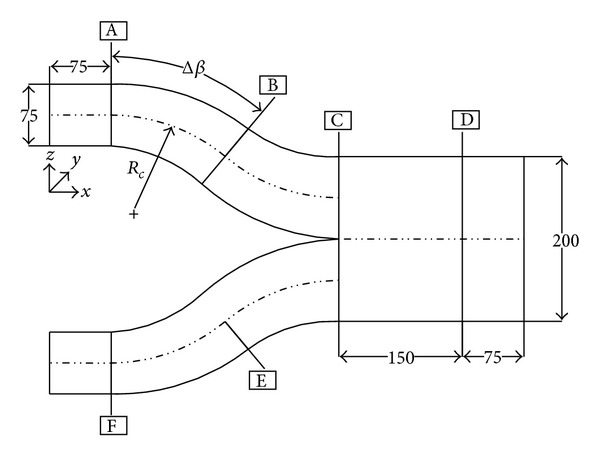
Schematic diagram of a twin air-intake.

The present study examines the effects of the two flow control techniques on the aerodynamic performance of a twin air-intake with turning angle (Δ*β*) of 20° as shown in Figure 1 along with the coordinate system. Vane-type VG being passive flow control device is shown in Figure 3, whereas VGJ as shown in Figure 4 is used for active flow control in the study. 

### 2.1. Twin Air-Intake Model

The schematic diagram of a twin air-intake (*R*_*c*_ = 420 mm, Δ*β* = 20°, *A*_*r*_ = 1.33) is shown in Figure 1. Planes A and F are rectangular inlets (75 × 75 mm^2^) while planes B and E are inflexion planes. Both of the S-shaped individual limbs are merged at plane-C and AIP is located 10 mm prior to the exit of the air-intake having cross-section of 75 × 200 mm^2^. A 75 mm long rectangular straight pipes are attached before inlets and beyond plane-D to reduce the atmospheric disturbances. 

### 2.2. Flow Control Devices

Two types of flow control devices—vane-type vortex generator (VG) and vortex generator jet (VGJ) are used in the study. VG is used for passive flow control and VGJ is used for active flow control in the air-intake.

#### 2.2.1. Vane-Type Vortex Generator

**Figure 2:**
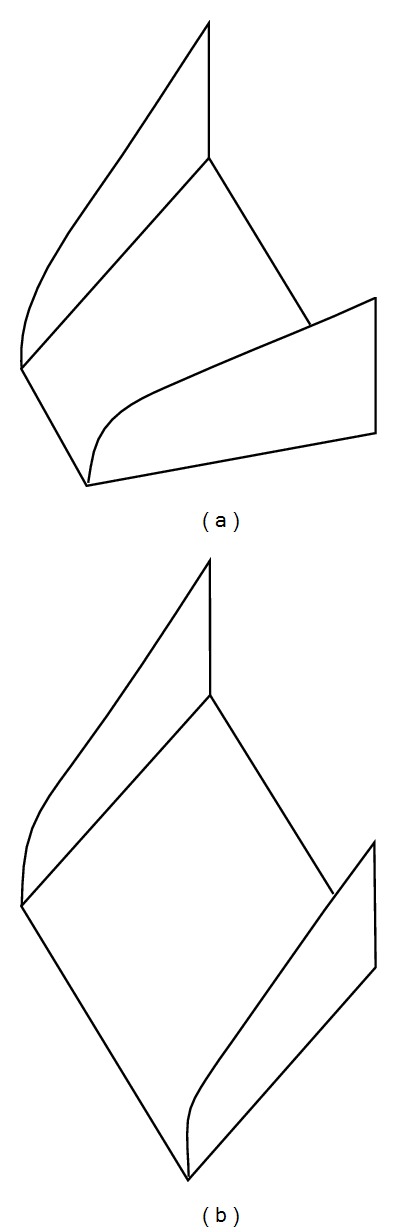
Vane-type vortex generators: (a) counter- and (b) corotating.

**Figure 3:**
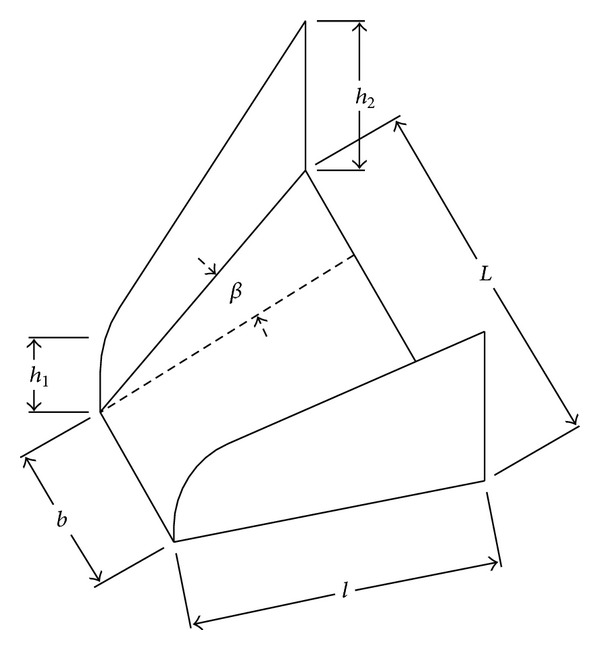
Nomenclature of a vane-type vortex generator (counterrotating).

The VG used in the study is designed as per Paul et al. [8], of trapezoidal shape, placed normal to the surface, and at various vortex generator angle (*β*). They are staggered as corotating (parallel) and counterrotating (V-shape) configurations as shown in Figure 2. The dimensions and nomenclature of the vane-type VG are shown in Figure 3. Two different VG sizes used in the study are referred to as VG-1 (smaller) and VG-2 (larger). The design parameters of these VGs are given below.

 VG-1: *β* = 13.5°, *b* = 6.0 mm, *h*_1_ = 2.0 mm, *h*_2_ = 4.0 mm, *L* = 11.0 mm, *ℓ* = 11.0 mm
 VG-2: *β* = 27.0°, *b* = 6.0 mm, *h*_1_ = 3.0 mm, *h*_2_ = 6.55 mm, *L* = 18.0 mm, *ℓ* = 13.2 mm.

#### 2.2.2. Vortex Generator Jet

**Figure 4:**
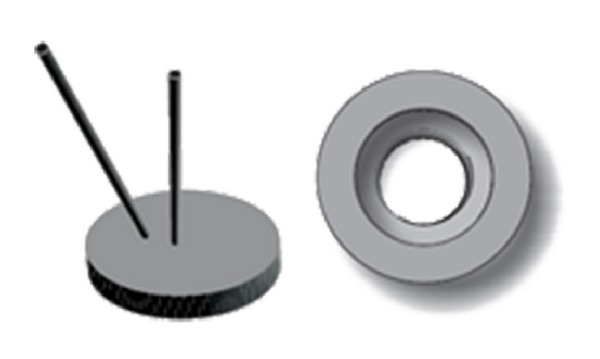
Vortex generator jet (VGJ).

Vortex generator jet (VGJ) is designed as given in the literature [11, 12] and is used for the study. Two stainless tubes of 2 mm diameter are provided at different pitch angles (0° and 45°) in the VGJ arrangement. The system is fitted in a 20 mm diameter rotating plug as shown in Figure 4, which enables to rotate it around 360° yaw angle. The jet was issued at a velocity ratio (i.e., jet velocity to free stream velocity of air) of 2.

### 2.3. Instrumentation

Digital micromanometer with a pressure scanner (make: Furness Controls, UK) is used to measure pressure, velocity when connected to a measuring instrument like pitot-static tube and wall-static pressure taps. A precalibrated five-hole static pressure probe with probe traverse system is used to carry out the steady-state measurements of three velocity components, inflow angles, static and total pressures simultaneously for a point in a flow field. A calibrated orifice meter (design as per ISO: 5167-2003) is used to provide the predetermined mass flow rate into the VGJ. Uncertainties associated in the experimentation are determined as per Kline [15] and are listed in Table 1.

## 3. Experimental Procedure

**Figure 5:**
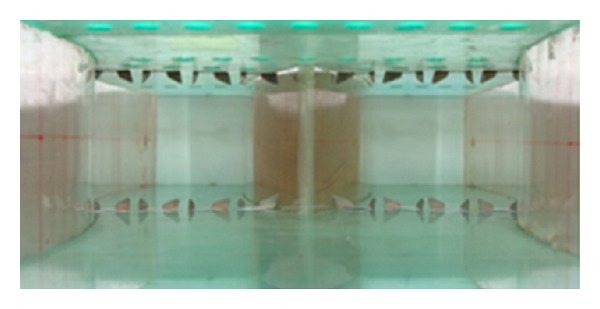
Location of VG (counterrotating) on the top-bottom walls of the air-intake.

Mass-averaged velocity of air is maintained at 20 m/s at both inlets (planes A and F) of twin air-intake. Additional air at a velocity of 40 m/s is issued through the VGJ connected at side walls of the air-intake. Both VG and VGJ are located at the inflexion planes (planes B and E) of individual limbs of the air-intake. VGs are attached to either top-bottom interior walls (eight on each of the two top and bottom walls) and side interior walls (three on each of the four side walls) of the air-intake as shown in Figure 5. The five different configurations of VG used in the study are as follows:

 Case-1: no VG.
 Case-2: counterrotating VG-1 array placed at top-bottom and side walls.
 Case-3: corotating VG-1 array placed at top-bottom and side walls.
 Case-4: counterrotating VG-2 array placed at top-bottom and side walls.
 Case-5: corotating VG-2 array placed at top-bottom and side walls.

**Figure 6:**
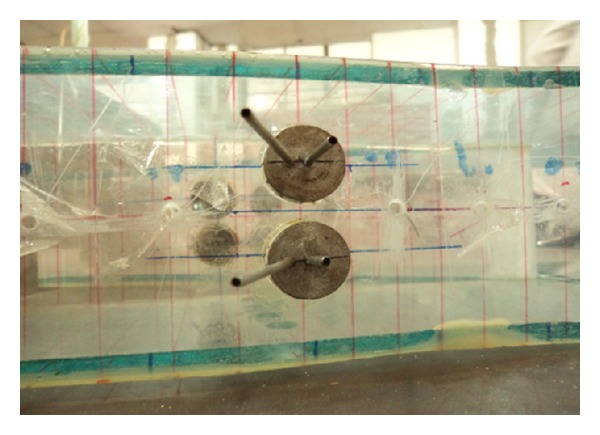
Location of VGJ on the side walls of the air-intake.

**Figure 7:**
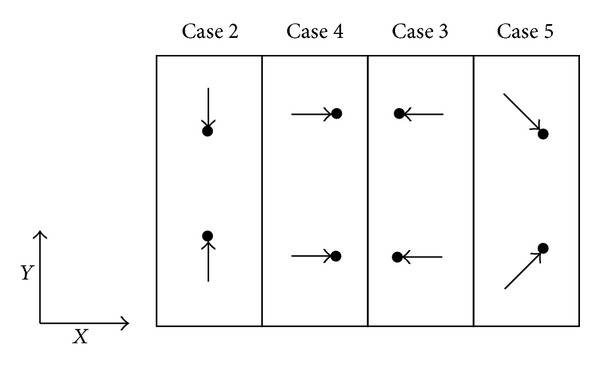
Different jet configurations of VGJ in the yaw (*x*-*y*) plane for pitch angle of 45°.

Likewise, two VGJs are affixed at each of the four interior side walls at the inflexion planes (B and E) of the air-intake as shown in Figure 6. Experiments were conducted for five various pitch and yaw combinations and are furnished below as well as depicted in Figure 7 for clarity. Study of the bare air-intake (i.e., without VG or VGJ) is referred to in the following sections as “Case-0.”

 Case-1: Pitch 90° (VGJs are directed perpendicular to the side walls).
 Case-2: Pitch 45° and yaw 90° (Jets facing each other).
 Case-3: Pitch 45° and yaw 180° both.
 Case-4: Pitch 45° and yaw 0° both.
 Case-5: Pitch 45° and yaw 45° (converging).

## 4. Results and Discussion

This section discusses the results obtained from experimentation using various flow control devices, namely, VG and VGJ.

### 4.1. Flow Control Using Vortex Generators (VGs)

Different combinations of VG are tested and their results are tabulated in Table 2. Both geometry (VG height *h*_2_ and VG angle) and locations of vane-type VG are varied to find out the optimum aerodynamics performance in twin air-intake. This type of VG arrays is used with the aim of manipulating the flow to reduce nonuniformity and total pressure distortion and possibly improve static pressure recovery by means of separation and secondary flow control. For this, two approaches are necessary. The first is to create a strong mixing between the boundary layer fluid and the main flow to produce a fairly uniform flow at the AIP. The second is to counter the effect of the secondary flows, which tend to accumulate the low energy boundary layer flow in one area, and redistribute the boundary layer evenly around the perimeter of the AIP, thus reducing the flow distortion [16].

It is found from the literatures [17, 18] that the vortex generators attached to the top and bottom walls of the air-intake effectively control the secondary flow instead of using it on side walls. This is evident from Table 2 that secondary flow non-uniformity (*S*_*io*_) sometimes increases for the air-intakes with vortex generators attached to its side walls, whereas the same parameter only records a reduction if vortex generators are attached to its top-bottom walls irrespective of its heights. 

Corotating VG array is useful in reducing flow separation if it is properly designed and located. The key advantage of co-rotating VG is their downstream effectiveness resulting in more effective usage of the vortex energy within the affected boundary layer. According to design wisdom, this type of VG has a few special advantages when used in twin air-intakes; namely: (a) the induced microvortices will remain close to the wall; consequently a “cleaner” core flow will result, and (b) the induced vortices will counteract the natural and often strong secondary flows, which can develop within the S-bend of each individual limb of such twin air-intakes. Counter-rotating VG array, on the other hand, has the disadvantages as compared to the co-rotating VG array, since the induced vortices tend to lift off the duct surface, thus reducing their effectiveness, causing higher loss in static pressure recovery and large total pressure distortion at AIP [19]. 

The use of co-rotating VG array is proved to be more successful as evident from Table 2. With effective use of such VG array, aerodynamic performance of nonfused air-intakes is improved in terms of *C*_PR_ (maximum rise by 6.2%), *C*_TL_ (maximum drop by 4.42%), DC_60_ (maximum reduction by 16.1%),and *σ*_*xo*_ (maximum reduction by 36.58%). From Table 2, co-rotating VG array when attached to the side walls of the air-intake offers the optimum performance. 

### 4.2. Flow Control Using Vortex Generator Jets (VGJs)

The VGJ technique is a time-varying control action to optimize performance under a wide range of flow condition. For VGJ, the strength of longitudinal vortices is controllable by varying the jet speed. The values of different performance parameters are given in Table 3 for all the cases of VGJ tested in the study. 

Jet dynamics is entirely controlled by vorticity. Generation of vortices at jet issuing source is completely dictated with convection in axial direction and diffusion in crossways. At the onset of instability in jets, amplitude of fluctuating vortices shoots up and subsequently proper mixing of layers starts. The vortices roll up and finally, the merging of these vortices in the freestream controls the amount of momentum transferred between layers, which consequently affects the point of separation. The sole purpose of using a VG or a VGJ is to have proper mixing between layers such that the layer close to the walls gains energy to continue and not to separate out. In order to control flow separation, it is therefore worthwhile to consider certain factors affecting the onset of instability, vortex roll up, and vortex merging in a jet. Moreover, when two VGJs at each side wall simultaneously issue jet at 40 m/s, the effect of orientation of two jets and their interaction play an important role. The factors which affect the flow separation are therefore (a) disturbances in the freestream, (b) disturbances due to boundary layers, wakes and small recirculation zones, and (c) the interaction between the two jets.

As in case of a twin air-intake, the VGJs are applied at the inflexion plane from the side walls; hence considering the above listed factors, there are freestream disturbances. As the flow negotiates a curved path introducing secondary flow and turbulence due to centrifugal effects, small recirculation zones could also be noticed towards the inner side of the air-intake. Hence, there are three vortex interactions possible, one between the free stream disturbances and the jet vortices, second between recirculation vortices, and jet vortices and third between the two jet vortices itself. This incident together with turbulence superimposed becomes highly complicated flow phenomena. Prediction of the correct behavior of VGJ in a flow stream is only possible during an experiment in a laboratory. Study the effect of jets at different orientation is also important. Johnston and Nishi [11] presented a similar studying of jet behavior downstream when they interacted with each other on a flat plate. The present study is, however, a little different due to the wall curvature.

Case-1 observes the effect of jet inserted perpendicular to the main flow stream through air-intake (i.e., pitch angle = 90°). The case proves to be best as it exhibits low total pressure loss and high static pressure recovery as shown in Table 3. This clearly indicates that all possible interaction of vortices shedding from the jets is quite capable of mixing layers and transferring momentum, hence energizing the near wall flow. Johnston and Nishi [11] performed interaction of different orientations at 45° pitch and various yaw angles and observed similar trends. 

When jets are oriented at a pitch angle of 45° and yaw angle of 90° (case-2), the jets converge the centerline. This case exhibits the least potential in terms of flow improvement and control and can be accounted to the fact that the vortex interaction produces momentum transfer but either insufficient or in reverse direction. The upper layer gets energized cascading energy from the near wall flow; hence, the separation point shifts upstream leading to higher pressure losses and lower static pressure recovery. This was exactly the behavior experienced by Johnston and Nishi [11] while using the converging jets.

Case-3 again includes jet oriented at a pitch angle of 45° and at a yaw angle of 180°; that is, the side view would show the jets aligned opposite to the flow. The case does not offer the best performance; however, reasonable improvement in performance parameters is observed. The results are well in agreement with the fact that when the jets are oriented at 180° to the main flow, two vortices streams are shed from each jet instead of one, though there are two jets; however, the performance parameters do not show the finest improvements for the reason that the two shed vortices are of relatively poorer strength and could not offer such an outcome that a single higher strength vortex could. The above is also true when jets are aligned with the flow at 0°; the case has been discussed under as case-4. Unlike case-3, the jets are now aligned to the direction of main flow keeping the jet orientation the same. This case shows slight improvement in the performance parameters as compared to the base value for the bare duct. Though being similar to case-3, nevertheless it does not show much improvement in performance as compared to case-3. The jets when aligned towards the flow shed two vortices of relatively low strength. But while the jets are aligned with the main flow direction, the interaction between the vortex and the free stream is not adequate since the vortices get carried away in the free stream relative to the amount of interaction occurred in case-1. It is, therefore, concluded that while the jets are aligned opposite to the main flow direction (case-3), the interaction between the flow and the vortices is adequately enough to provide an enhancement in performance.

In case-5, the jets are oriented at pitch and yaw angle both being 45°; that is, the jets look like diverging along the longitudinal mid-symmetry plane of the air-intake. The case shows improvement in flow parameters, the static pressure recovery being highest but accompanied with relatively higher total pressure loss that makes the case less effective as compared to the best case (case-1).

It is, therefore, emerged from the above discussion that the VGJ when directed perpendicular to the air-intake side walls (case-1) offers the best aerodynamic performance.

### 4.3. Performance Comparison of Different Flow Control Techniques

**Figure 8:**
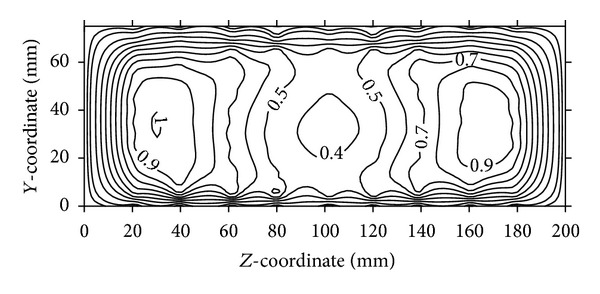
Normalized velocity contours at the outlet plane of bare air-intake.

**Figure 9:**
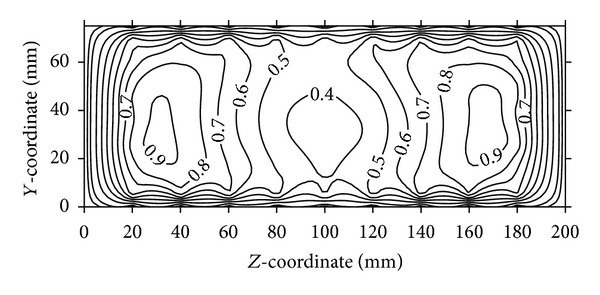
Normalized velocity contours at the outlet for air-intake (best VG case).

**Figure 10:**
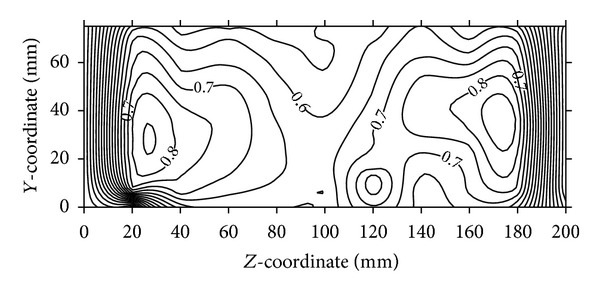
Normalized velocity contours at the outlet for air-intake (best VGJ case).

A number of combinations are tested using flow control devices:VG and VGJ and their comparison in terms of normalized velocity contours are presented in Figures 8–10. Velocity components are calculated from the pressure data taken by five-hole pressure probe using calibration charts. For the sake of brevity, the details of calculation are not however included here. Velocity contours are drawn using graphic software (Surfer) and the spatial velocity data are interpolated using kriging technique to draw the velocity contours, which are detailed in Stein [20]. 

Figure 8 shows the velocity contours at the outlet plane (plane-D) of a bare air-intake (without any VG or VGJ). The values of local velocities are normalized by the free-stream velocity available at the inlets of the air-intake. The velocity contours at the outlet of the duct clearly indicate that the peak velocities occur near the side walls and low velocity core remains at the centre. From Figure 9, it is clear that the two velocity peaks on either side of the air-intake are reduced to an extent due to the use of VG, whereas the weak core flow still exists. However, a noticeable change in the flow pattern is observed in Figure 10, when VGJ is used in the air-intake. The weak core flow is disappeared as there is an increase in velocity in the middle of the outlet plane, whereas the velocity peaks near the two side walls are reduced to a large extent indicating the flow uniformity at the outlet plane of the air-intake. As a result, both the secondary and axial flow non-uniformities are reduced to a great extent (30% and 13%, resp.) as shown in Table 4. 

Overall, the flow control of air-intake with VGJ promotes the highest static pressure recovery coefficient (*C*_PR_), lowest total pressure loss coefficient (*C*_TL_), maximum reduction of secondary flow non-uniformity (*S*_*io*_), and axial flow non-uniformity (*σ*_*xo*_). However, vane-type VG ensures minimum total pressure distortion coefficient (DC_60_) as compared to the air-intake with VGJ. In cases where VGJ is used, the extra mass flow injected through it helps in decreasing the pressure losses. 

## 5. Conclusions

Experimental studies on air-intake diffuser are conducted and various combinations of vane-type vortex generator (VG) and vortex generator jet (VGJ) are tested. The following conclusions are drawn from the current study.

- Comparison between the two flow control techniques shows that the use of VGJ is more effective as compared to VG. It is observed that VGJ has the potential to change the flow pattern drastically as compared to VG. Furthermore, for flow situations where stall control is not needed, parasitic drag can be avoided with the jet flow turned off. On the contrary, vane-type VG is always exposed in the flow and can increase drag. The VGJ technique accomplishes flow separation control only when it is necessary and therefore it is favored over vane-type VG for both design and off-design conditions.
- In case of vortex generator jets, the best result is given when the jet is directed perpendicular to the side walls at a pitch angle of 90°. Two most important performance parameters—static pressure recovery is increased by 7.8% and total pressure loss is reduced by 40.7% as compared to all other cases of VGJ.
- In case of vane-type vortex generators, the best result is given when co-rotating VG-2 (big-sized VG) is attached to the side walls of the air-intake. In this case, static pressure recovery is increased by 5.3%, but total pressure loss is reduced by only 4.5% as compared to all other cases of VG.

## Figures and Tables

**Table 1:** Least count and uncertainty in the measured parameters.

Parameter	Symbol	Instrument	Least count	Uncertainty used
Vertical traverse	*y*	Probe traversing mechanism	0.1 mm	±0.1 mm
Angle	*α*, *β*	Probe orientation mechanism	1°	±1°
Pressure	*p*	Digital micromanometer	0.001 N/m^2^	±0.025% of full scale deviation
Ambient temperature	*T*	Digital thermometer	0.5°C	±0.5°C

**Table 2:** Values of performance parameters for all the cases of VG.

VG sequence	Location	*C*_PR_	*C*_TL_	DC_60_	*S*_*io*_	*σ*_*xo*_
No VG (Case-0)	—	0.452	0.113	0.259	0.010	5.420
VG-1 (Counter)	Side walls	0.473	0.120	0.253	0.011	5.359
	Top-bottom	0.456	0.112	0.248	0.011	5.270
VG-1 (Corot.)	Side walls	0.473	0.119	0.249	0.011	5.374
	Top-bottom	0.459	0.112	0.223	0.011	5.208
VG-2 (Counter)	Side walls	0.480	0.118	0.232	0.011	4.972
	Top-bottom	0.459	0.113	0.237	0.017	3.593
VG-2 (Corot.)	Side walls	0.476	0.108	0.228	0.013	5.370
	Top-bottom	0.461	0.112	0.217	0.017	3.438

**Table 3:** Values of performance parameters for all the cases of VGJs.

Parameter	Case-0	Case-1	Case-2	Case-3	Case-4	Case-5
*ζ*	0.68	0.74	0.67	0.72	0.69	0.74
*C*_TL_	0.113	0.067	0.114	0.085	0.095	0.090
*S*_*io*_	0.010	0.007	0.008	0.007	0.009	0.010
*σ*_*xo*_	5.420	4.695	5.510	5.215	4.551	5.024
DC_60_	0.218	0.236	0.209	0.272	0.168	0.251

**Table 4:** Performance comparison of air-intake using flow control.

Flow control	*C*_PR_	*C*_TL_	DC_60_	*S*_*io*_	*σ*_*xo*_
Bare air-intake	0.452	0.113	0.218	0.010	5.420
With VGJ	0.487	0.067	0.236	0.007	4.695
With VG	0.476	0.108	0.174	0.013	5.370

## Nomenclature

*A*: Cross-sectional area … mm
*A*_*r*_: Area ratio, *A*_*e*_/*A*_*i*_
*C*_PR*i*_: Ideal static pressure recovery coefficient, *C*_PR*i*_ = 1 − (1/*A*_*r*_)^2^
*C*_PR_: Actual static pressure recovery coefficient, *C*_PR_ = (*p*_*s*_ − *p*_*si*_)/((1/2)*ρU*_av*i*_^2^)
*C*_TL_: Total pressure loss coefficient, *C*_TL_ = (*p*_*ti*_ − *p*_*t*_)/((1/2)*ρU*_av*i*_^2^)
DC_60_: Distortion coefficient, DC_60_ = (*p*_*te*_ − *p*_*t*__60_)/((1/2)*ρU*_av*e*_^2^)
*p*_*s*_: Static pressure
*p*_*t*_: Total pressure at outlet
*p*_*t*60_: Total pressure at worst 60° sector
*R*_*c*_: Radius of curvature
*S*_*io*_: Secondary flow non-uniformity, *S*_*io*_ = ∑*U*_*yz*_/*n* × *U*_av*i*_
*U*_av_: Average velocity
*V*: Velocity component
*β*: Vortex generator angle
*δ*: Boundary layer thickness
Δ*β*: Turning angle of air-intake
*σ*_*xo*_: Axial flow non-uniformity index: $\sigma_{xo}=\sqrt{\sum {\left(U_{x}-U_{x\text{av}}\right)}^{2}/n}$
*ζ*: Effectiveness of air-intake, *ζ* = *C*_PR_/*C*_PR*i*_.

### Subscript

av: Average
*e*: Exit
*i*: Inlet
*x*: Component in *x*-direction
*yz*: Cross-component.

## References

[1] Seddon J (1988). *Introduction To Intake Aerodynamics*.

[2] Lin JC, Selby GV, Howrad FG (1991). Exploratory study of vortex-generating devices for turbulent flow separation control. *AIAA Paper No.*.

[3] Lin JC (1999). Control of turbulent boundary layer separation using micro-vortex generators. *AIAA Paper No.*.

[4] Bernard A, Foucaut JM, Dupont P, Stanislas M (2003). Decelerating boundary layer: a new scaling and mixing length model. *AIAA Journal*.

[5] Jenkins L, Gorton SA, Anders S (2002). Flow control device evaluation for an internal flow with an adverse pressure gradient. *AIAA Paper No.*.

[6] Lin JC (2002). Review of research on low-profile vortex generators to control boundary-layer separation. *Progress in Aerospace Sciences*.

[7] Reichert BA, Wendt BJ (1993). An experimental investigation of S-duct flow control using arrays of low profile vortex generator. *AIAA Paper No.*.

[8] Paul AR, Ranjan P, Patel VK, Jain A (2013). Comparative studies on flow control in rectangular S-duct diffuser using submerged-vortex generators. *Aerospace Science and Technology*.

[9] Paul AR, Ranjan P, Upadhyay RR, Jain A Passive flow control in twin air-intakes.

[10] Paul AR, Ranjan P, Upadhyay RR, Jain A (2011). Passive flow control in twin air-intakes. *World Academy of Science, Engineering and Technology*.

[11] Johnston JP, Nishi M (1990). Vortex generator jets. Means for flow separation control. *AIAA Journal*.

[12] Johnston JP, Mosier BP, Khan ZU (2002). Vortex generating jets; effects of jet-hole inlet geometry. *International Journal of Heat and Fluid Flow*.

[13] Sullerey RK, Pradeep AM (2002). Effectiveness of flow control devices on S-duct diffuser performance in the presence of inflow distortion. *International Journal of Turbo and Jet Engines*.

[14] Harrison NA, Anderson J, Fleming JL, Ng WF (2007). Experimental investigation of active flow control of a boundary layer ingesting serpentine inlet diffuser. *AIAA*.

[15] Kline SJ (1985). The purposes of uncertainty analysis. *Journal of Fluids Engineering, Transactions of the ASME*.

[16] Anabtawi AJ, Blackwelder RF, Lissaman PBS, Liebeck RH (1999). An experimental study of vortex generators in boundary layer ingesting diffusers with a centerline inlet. *AIAA Paper No.*.

[17] Sullerey RK, Mishra S, Pradeep AM (2002). Application of boundary layer fences and vortex generators in improving performance of S-duct diffusers. *Journal of Fluids Engineering, Transactions of the ASME*.

[18] Sullerey RK, Pradeep AM (2004). Secondary flow control using vortex generator jets. *Journal of Fluids Engineering, Transactions of the ASME*.

[19] Anderson BH, Huang PS, Paschal WA, Cavatorta E (1992). A study on vortex flow control of inlet distortion in the re-engined 727-100 center inlet duct using computational fluid dynamics. *AIAA Paper No.*.

[20] Stein ML (1999). *Statistical Interpolation of Spatial Data: Some Theory For Kriging*.

